# Adherence to the Australian dietary guidelines and development of depressive symptoms at 5 years follow-up amongst women in the READI cohort study

**DOI:** 10.1186/s12937-020-00540-0

**Published:** 2020-04-10

**Authors:** Rachelle S. Opie, Kylie Ball, Gavin Abbott, David Crawford, Megan Teychenne, Sarah A. McNaughton

**Affiliations:** Institute for Physical Activity and Nutrition, School of Exercise and Nutrition Sciences, Faculty of Health, Deakin University, 221 Burwood Highway, Burwood, VIC 3125 Australia

**Keywords:** Diet, Australian dietary guideline index, Depression, Women, Socioeconomic disadvantage

## Abstract

**Background:**

Depression is the single largest contributor to global disability. There is growing evidence that a healthy diet is associated with reduced depression risk. However, beyond the Mediterranean diet, few longitudinal studies have explored the relationship between adherence to national dietary guidelines and depression. Hence, this study investigates the relationship between adherence to Australian Dietary Guidelines and depressive symptoms.

**Methods:**

Data was drawn from the READI longitudinal study, a prospective cohort study of socioeconomically disadvantaged Australian women. This analysis includes a sub-sample of 837 women. A generalized linear model was used to explore whether baseline diet (assessed using the Dietary Guideline Index (DGI-2013; score range 0 to 85)) was associated with risk of developing depressive symptoms (measured by the Centre for Epidemiologic Studies Depression (CES-D)) at 5 years follow-up, whilst adjusting for potential confounders. A fixed-effects model was used to assess associations between concurrent changes in diet quality and depressive symptoms from baseline to 5 years follow-up.

**Results:**

An association between baseline diet quality and risk of developing depressive symptoms at follow-up was observed, where a 10 unit increase in DGI-2013 score was associated with an estimated 12% lower risk of developing heightened depressive symptoms (RR = 0.875, 95%CI 0.784 to 0.978, *p* = 0.018). The fixed-effects model indicated that an increase in DGI score over 5 years follow-up was associated with a lower (improved) CES-D score (B = -0.044, 95% CI − 0.08 to − 0.01, *p =* 0.024).

**Conclusions:**

Our results provide evidence that better adherence to the Australian Dietary Guidelines may result in improved depressive symptoms. The growing high-quality evidence regarding the diet-depression relationship provides us with a rationale for developing strategies for supporting dietary behaviour change programs to lower depression rates.

## Introduction

Depression is ranked as the single largest contributor to global disability [1, 2]. Internationally, over 300 million people are estimated to suffer from depression, equivalent to 4.4% of the world’s population [1]. Depression is more common amongst women than men [3], and amongst those of a low socio-economic position (SEP) (e.g. low education or income) [4]. It can lead to substantial distress, isolation of, and discrimination against, those affected [5], and is a major contributor to suicide deaths [1]. The global cost of mental illness such as depression has been estimated at US$2.5 trillion annually [6], which includes direct costs associated with diagnosis and treatment in the healthcare system, and indirect costs associated with income loss, lost productivity and labour force participation [5, 6].

Because compliance with (and efficacy of) antidepressant medication is commonly low [7, 8], with potentially undesirable side effects [9], exploration into the role of alternative strategies for the prevention and management of depression is important. An unhealthy diet is one of the major modifiable behavioural risk factors for mental disorders [10]. In recent years, research on diet-disease links has shifted its focus away from individual nutrients or single foods, and towards dietary patterns [11]. This approach is likely superior as it is reflective of the way people actually consume food, and allows for the exploration of the synergistic interactions of the nutrients and foods in combination. There is a growing body of evidence on the relationship between dietary patterns and depression, with studies showing that adherence to a high-quality diet is associated with reduced depression risk [12–16]. Only recently, randomized controlled trials (RCTs) have assessed this relationship, demonstrating associations between Mediterranean-type dietary patterns and reduced depressive symptoms, strengthening the evidence base regarding the link between diet and depression [17–19]. However, beyond the Mediterranean diet, few longitudinal studies have specifically sought to explore the relationship between adherence to the national dietary guidelines and depression [16].

The RCT evidence to date has shown how adherence to a healthy diet (e.g. rich in vegetables, fruit, nuts, legumes, seeds, and fish, and little or no packaged, highly processed foods high in added sugar, refined carbohydrates, and unhealthy fats [20]) may ameliorate existing depression, whereas prospective studies can provide insight into whether a healthy diet can prevent onset of future depression/depressive symptoms. Therefore, this study investigates the relationship between adherence to the Australian Dietary Guidelines and depressive symptoms amongst socioeconomically disadvantaged Australian women. While not targeting a specific disease, the dietary guidelines are intended for promoting good health, which includes good mental health. Yet, there remains limited evidence as to whether the guidelines are sufficient for achieving this. Hence, this study is important as this information could help inform development of new (or refinement of current) dietary guidelines for good mental health.

We hypothesise that women with a higher quality diet (e.g. higher adherence to the Australian Dietary Guideline Index (DGI-2013, 6)) will have a reduced risk of developing depressive symptoms at 5 years follow-up, compared to women with poorer quality diets (e.g. lower adherence to the DGI-2013). This hypothesis was derived in consideration of the fact that the Australian Dietary Guidelines constitute a healthy diet comprised of foods that are believed to be associated with health benefits for mood and depression [16, 20].

## Materials and methods

### Study

This study investigates the longer-term associations of usual dietary intake (assessed according to adherence to the Australian Dietary Guidelines) and development of depressive symptoms (at 5 years follow-up) amongst socioeconomically disadvantaged Australian women. Data was drawn from the Resilience for Eating and Activity Despite Inequality (READI) longitudinal study, a prospective cohort study of 4349 women aged 18–46 years recruited randomly using the electoral roll from 80 socio-economically disadvantaged urban and rural neighborhoods of Victoria, Australia [21]. The aims of the READI study were to investigate pathways (personal, social and structural) by which socio-economic disadvantage influences obesity and its behavioural risk factors (physical inactivity, poor diet), and to explore mechanisms underlying ‘resilience’ to obesity risk in socio-economically disadvantaged women and children. Key outcomes include BMI, and survey data were collected on hypothesised personal, social and perceived environmental predictors of body weight. Detailed methods are provided elsewhere [21]. The study was approved by the Deakin University Human Research Ethics Committee (approval number 2006–091). Women provided written, informed consent to participate.

In late 2007 and early 2008, a total of 4934 women (45% response rate) completed a mailed survey at baseline. Of those who responded, 585 women were excluded for the following reasons: not currently residing in one of the selected study neighborhoods (*n* = 571), outside the valid age range (or missing age data) (*n* = 9), not the intended participant (survey was not completed by the women it was addressed to) (*n* = 3), or having withdrawn from the study (*n* = 2). Thus, 4349 women remained who were eligible to participate. Of this cohort, those who consented to further follow-up and remained eligible (*n* = 3019 women) were re-contacted to complete a follow-up survey 3 years after the baseline survey (2010–11). This survey was completed by 1913 women. A further follow-up survey 5 years after the baseline survey (2012–13) was completed by 1560 women (36% of initial cohort). Data at 3 years follow-up was not used in this present analysis. Only baseline and 5 year follow-up data points were used as this study focused on the longer-term associations of usual dietary intake with development of depressive symptoms. Importantly, supplementary analyses (data not shown) demonstrated that minimal dietary changes occurred over this 5 year time frame (from baseline to 5 year follow-up), with a mean change of 0.67 DGI points.

**Fig. 1:**
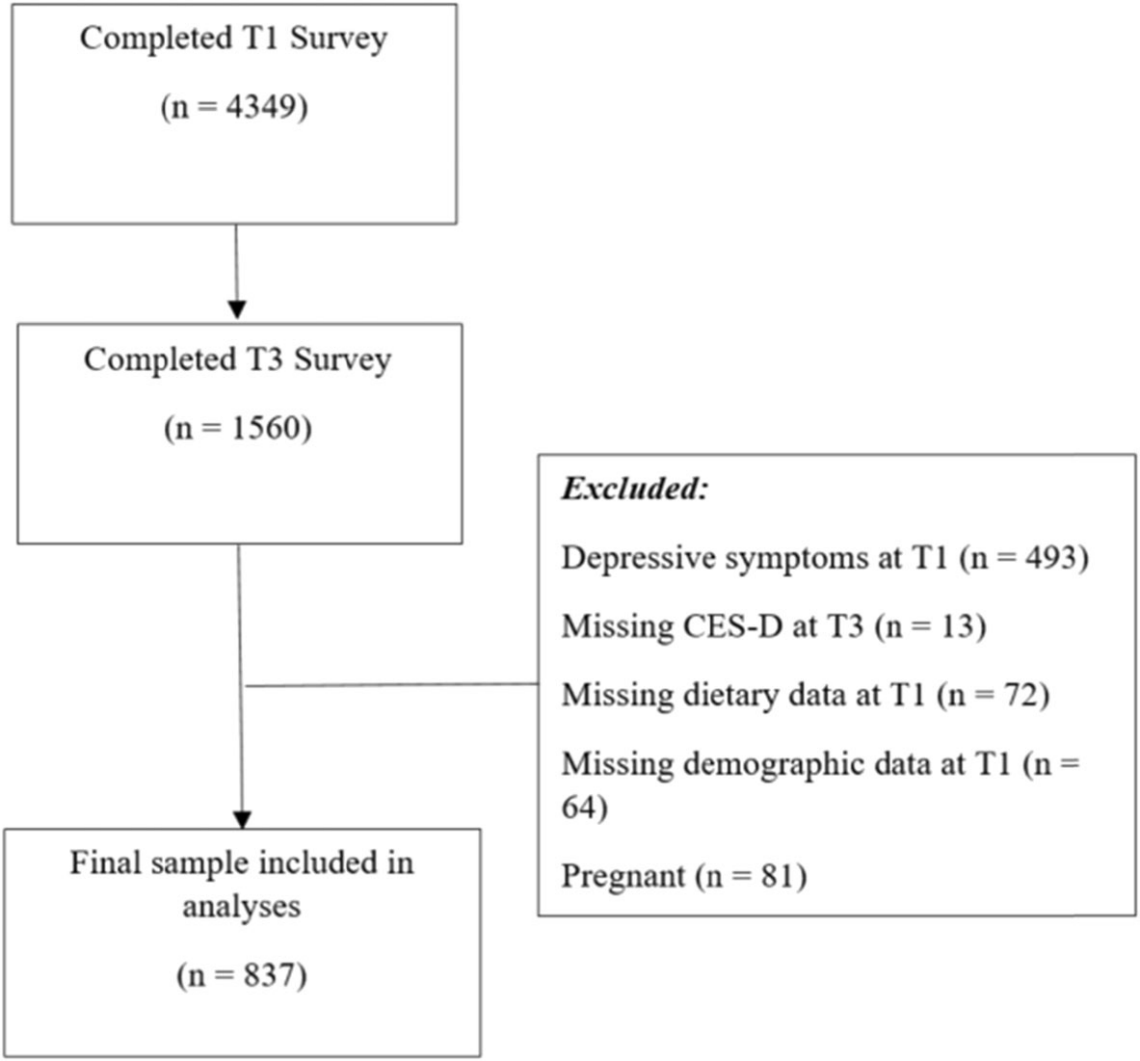
Flowchart of inclusion and exclusion into the study

In an effort to reduce the possibility of reverse causality, women with reported depressive symptoms (Centre for Epidemiologic Studies Depression (CES-D) ≥ 10) at baseline were excluded from analyses (*n* = 493). Since development of depressive symptoms was the key outcome, women were also excluded if they had not completed the CES-D at 5 years follow-up (*n* = 13). Furthermore, women who had not completed the dietary questionnaire at baseline (*n* = 72), or who had missing demographic and anthropometric information (*n* = 64) were excluded. Because dietary behaviours (and requirements) typically alter in pregnancy [22], and are therefore not reflective of habitual intake, pregnant women (at any time point) (*n* = 81) were not included. Thus, of the total possible baseline and 5 year follow-up completers (*n* = 1560), 837 women remained in the study for analyses. Refer to Fig. 1 for flowchart of inclusion and exclusion into the study.

### Primary outcome

#### Depressive symptoms

Depressive symptoms were assessed using the 10-item Center for Epidemiologic Studies Depression Scale (CES-D) [23] at baseline and 5 years follow-up. This scale is a commonly-used self-reported measure of depressive symptoms with good retest reliability and predictive validity compared with the original 20-item version [23–27]. Responses were reported using a four-point Likert scale ranging from ‘rarely’ (scored 0) to ‘most of the time’ (scored 3). The total score was derived by calculating the sum of 10 items (possible scores range from 0 to 30), with higher scores indicating more depressive symptoms. Depressive symptoms were also presented as a categorical variable with a score of ≥10 used to define risk of heightened depressive symptoms [23, 24]. This cutoff value has been shown to produce good test-retest reliability [23, 24].

### Predictor/exposure measures

#### Dietary assessment

At baseline and five-year follow-up, a semi-quantitative short food frequency questionnaire (FFQ) was used to assess frequency of consumption of vegetables, fruits, grain (cereal) foods, meat and alternatives, dairy products, discretionary foods, added sugars, fruit juice, soft drink, and alcoholic beverages over the previous month. This paper-based mailed survey questionnaire was completed via self-report, and was constructed using several previously published and validated Australian nutrition surveys [28–30]. For example, questions on fruit were sourced from the 1995 National Nutrition Survey (NNS), and questions on grain (cereal) foods were sourced from the Cancer Council of Victoria FFQ, which have been validity-tested in a wide range of population groups [31, 32]. Table 1 provides details of the survey questions and response options for each food group. For the analyses, frequencies were converted to daily equivalents. Unless specified, serve sizes were based on Australian Guide to Healthy Eating (AGHE) values. The FFQ also included questions on how often fat was usually trimmed from meat and the type of milk and bread usually consumed.

**Table 1:** Survey questions and response options for each food group

Food Group	Survey Question	Number of response options	Response options
**Vegetables**	About how many serves of vegetables do you usually eat per day?	8	“I don’t eat vegetables” to “6 serves or more/day”
	About how many serves of potatoes do you usually eat per week?	8	“I don’t eat potatoes” to “6 serves or more/week”
**Fruit**	About how many serves of fruit do you usually eat per day?	8	“I don’t eat fruit” to “6 serves or more/day”
**Grain (cereal) foods** (bread; pasta, rice, noodles; breakfast cereal)	About how many slices of bread do you usually eat per day?	8	“I don’t eat bread” to “8 slices or more/day”
	In the past month, about how often have you had the following?	9	“Never or less than once/month” to “6 or more times a day”
**Meat and alternatives** (red meat; chicken; fish; dried beans/peas; eggs; nuts)	In the past month, about how often have you had the following?	9	“Never or less than once/month” to “6 or more times a day”
**Dairy** (cheese; yoghurt; milk; flavoured milk)	In the past month, about how often have you had the following?	9	“Never or less than once/month” to “6 or more times a day”
	About how many serves of plain milk in total do you usually drink each day?	9	“I don’t drink plain milk” to “10 or more serves/day”
	About how much flavoured milk in total do you usually drink each day?	9	“I don’t drink flavoured milk” to “10 or more serves/day”
**Discretionary foods** (hot chips; potato crisps, salty snacks; cake doughnuts, sweet biscuits; pies, pastries, sausage rolls; fast foods; pizza; meat products)	About how many serves of hot chips, French fries, wedges or fried potatoes do you usually eat per week?	8	“I don’t eat chips” to “6 serves or more/week”
	In the past month, about how often have you had the following?	9	“Never or less than once/month” to “6 or more times a day”
**Added sugars** (chocolate, lollies; soft drink; fruit juice)	In the past month, about how often have you had the following?	9	“Never or less than once/month” to “6 or more times a day”
	About how much soft drink do you usually drink each day?	9	“I don’t drink soft drink” to “10 or more serves/day”
	About how much fruit juice do you usually drink each day?	9	“I don’t fruit juice” to “10 or more serves/day”
**Alcohol**	On days when you were drinking alcohol, about how many glasses of beer, wine and/or spirits altogether did you usually drink?	11	“I don’t drink alcohol” to “10 or more glasses/day”

#### Diet quality

Diet quality, reflecting compliance with dietary guidelines and consumption of the total diet / overall dietary pattern was assessed. A modified version of the Dietary Guideline Index (DGI-2013) [33], which reflects adherence to the Australian Dietary Guidelines [34] was used. The DGI-2013 has been shown to be a valid measure of diet quality in the Australian population, and is associated with intakes of key nutrients and health outcomes [33, 35–37]. The DGI-2013 reflects age- and sex-specific recommendations for the consumption of the five key food groups (vegetables, fruit, grain (cereal) foods, lean meat and alternatives, and dairy) which comprise the AGHE [34]. Discretionary items (e.g. cakes, processed meats, confectionary, pastries, chips, fried foods, soft drinks, alcohol), which are high in energy, saturated fat, sugars, salt and/or alcohol, and hence not an essential or necessary part of the diet, are also included in the DGI-2013. The original DGI-2013 consists of 13 components [33], with a total score range from 0 to 130. However, the DGI was modified for use in this study because adequate measures of 1. Food variety; 2. Proportion of lean meats and alternatives to total meat and alternatives per day; 3. Water intake; 4. Unsaturated oils, fats or spreads; and 5. Salt intake could not be calculated from the FFQ used in this study.

Supplementary Table 1 (see Additional file 1) provides details of the nine components that comprise this modified version of the DGI. Eight components of the DGI were scored out of ten, with zero indicating the guideline was not met and ten indicating the guideline was achieved. Lean meat and alternatives was the only exception, and was scored out of five (consistent with the original DGI) [36]. Cut-offs for minimum and maximum scores are provided in supplementary Table 1, and individuals who consumed intermediate amounts were scored proportionately. The total score ranged from 0 to 85, with a higher score indicating greater compliance with the dietary guidelines, and hence a better diet quality. Women were considered to have “met” each guideline if the maximum possible DGI-2013 score of 10 (with the exception of lean meat and alternatives which has a maximum DGI-2013 score of 5) was achieved for that DGI component. Diet quality, using the modified DGI-2013, was presented as a continuous score. Additionally, intake of individual food groups by READI participants was reported, to allow for comparisons with the AGHE recommendations and Australian population norms.

### Potential confounders

Confounders were selected a-priori based on existing evidence that these variables have a potential to influence both diet and depression [38]. All confounders were measured at baseline, and were as follows: age (continuous); highest education level (low (less than high school), medium (high school/trade/diploma), high (tertiary)); country of birth (Australia, overseas); employment status (full-time, part-time, not currently employed); relationship status (married/de facto, separated/divorced/widowed, never married); location of residence (urban, rural); smoking status (never smoked, former smoker, occasional smoker, regular smoker); BMI (based on self-reported height and weight and categorised as under/healthy weight, overweight, obese according to WHO cut-points [39]), and leisure-time physical activity, assessed using the International Physical Activity Questionnaire (IPAQ) [40]. Total leisure-time physical activity represented the weekly sum of the number of minutes spent in vigorous- and moderate- intensity leisure-time physical, and walking for leisure. This domain-specific physical activity (i.e. leisure-time) was included as it has the strongest association with mental health and mental ill-health (see meta-analysis by White et al., 2017 [41]), whereas evidence regarding the link between other domains of physical activity (e.g. occupational, domestic and transport-related physical activity) and mental health/ill-health is less consistent and/or suggests null associations.

### Statistical analysis

Analyses were conducted using Stata/SE Version 15.0 (StataCorp, College Station, TX). The significance level was set at *P* < 0.05. Baseline characteristics of the final eligible sub-sample included in this analysis (*n* = 837) were compared to those of the remaining (excluded) READI sample (*n* = 3512). Baseline characteristics were also described for women according to reporting of depressive symptoms at 5 years follow-up. When comparing the two groups Chi-square tests were used for categorical variables. Independent samples t-tests were performed for continuous variables, or Mann-Whitney U Test as the non-parametric alternative.

A generalized linear model was used to explore whether baseline diet was associated with risk of developing depressive symptoms at 5 years follow-up, whilst adjusting for potential confounders. For this model, the Poisson family with a log link was specified in order to obtain Risk Ratios (RR) for exposure variables [42]. Baseline diet quality (DGI-2013) (included as the exposure variable) was analyzed as a continuous variable. The model included robust standard errors adjusted for clustering within neighborhoods. Associations between concurrent change in diet (DGI score) and depressive symptoms (CES-D score) were assessed from baseline to 5 years follow-up using fixed-effects models. This analysis allows for estimation of subject-specific associations in longitudinal studies, and examines within-individual changes accounting for any time-invariant confounding since each participant acts as her own control. This model included all women with DGI and CES-D data at baseline and 5 year follow-up (both with and without reported depressive symptoms at baseline), who met the inclusion criteria (*n* = 1092).

## Results

Comparing the final eligible sub-sample (*n* = 837) and the excluded READI sub-sample (*n* = 3512), the eligible sub-sample participants had lower levels of baseline depressive symptoms (as expected based on key exclusion criteria), were older, and were more likely to be Australian born, married, reside in a rural setting, have completed tertiary education, and less likely to be unemployed (Table 2). Additionally, the eligible sub-sample participants had a higher adherence to Australian dietary guidelines, higher leisure-time activity levels, and were less likely to be obese or smoke compared to the excluded sub-sample.

**Table 2:** Baseline characteristics of the final eligible sub-sample included in this analysis compared to the excluded READI sample and Victorian / Australian level SEP data

	Included eligible sub-sample (***n*** = 837)	Excluded READI sub-sample (***n*** = 3512)	*P*	Victorian / Australian level SEP data [43]
Depression (CES-D), mean (SD)	4.70 (2.55)	9.23 (5.68)	***p*** **< 0.0005**	*–*
DGI, mean (SD)	53.78 (11.38)	50.56 (12.07)	***p*** **< 0.0005**	*–*
Age, mean (SD)	37.10 (7.29)	33.79 (8.20)	***p*** **< 0.0005**	*–*
Highest education level, % (*n*)				
Low (less than high school)	21.4 (179)	22.3 (767)	***p*** **< 0.0005**	–
Medium (high school /trade/diploma)	46.8 (392)	52.9 (1824)		–
High (tertiary)	31.8 (266)	24.8 (854)		29.5%
Born in Australia, % (*n*)	91.9 (769)	88.2 (3082)	***P*0.003**	73.8%
Employment status, % (*n*)				
Full-time	37.3 (312)	38.3 (1301)	***p*** **< 0.0005**	38.4%
Part-time	35.5 (297)	27.9 (948)		35.8%
Not currently employed	27.2 (228)	33.7 (1144)		
Relationship status, % (*n*)				
Married/de facto	75.0 (628)	63.2 (2201)	***p*** **< 0.0005**	56.0%
Separated/divorced/widowed	7.6 (64)	8.8 (306)		–
Never married	17.3 (145)	28.1 (978)		–
Area of residence, % (*n*)				
Urban	40.6 (340)	47.7 (1676)	***p*** **< 0.0005**	–
Rural	59.4 (497)	52.3 (1836)		–
Smoking status, % (*n*)				
Never smoked	54.0 (452)	49.4 (1732)	***p*** **< 0.0005**	56.6%
Former smoker	27.6 (231)	23.8 (835)		21.2%
Current smoker	18.4 (154)	26.9 (942)		–
BMI categories, % (*n*)				
Under/healthy weight	55.6 (465)	52.0 (1688)	***p*** **< 0.0005**	54.8%
Overweight	27.6 (231)	24.8 (806)		26.5%
Obese	16.8 (141)	23.1 (751)		18.7%
BMI, mean (SD)	25.64 (5.42)	26.21 (6.20)	*P* = 0.159	*–*
Total minutes per week leisure time physical activity, mean (SD)	221.17 (288.29)	208.88 (313.46)	***P*** **< 0.0005**	–

Women with depressive symptoms at 5 years follow-up were more likely to smoke, and/or have an obese BMI at baseline, than women without depressive symptoms at follow-up. Additionally, baseline diet quality was slightly better amongst individuals without depressive symptoms at follow-up, with a mean difference of 2.83 points on the DGI (mean (SD) 51.54 (11.12) with depressive symptoms, 54.37 (11.39) without depressive symptoms). The groups were found to be similar for the remaining baseline characteristics. Refer to Table 3 for details.

**Table 3:** Baseline characteristics of individuals with and without depressive symptoms at 5 years follow-up

Baseline characteristic	Depressive symptoms at 5 years follow-up *n* = 174	No depressive symptoms at 5 years follow-up *n* = 663	***P***
Depression (CES-D), mean (SD)	5.93 (2.24)	4.38 (2.54)	***P*** **< 0.0005**
DGI score, mean (SD)	51.54 (11.12)	54.37 (11.39)	***P*** **= 0.003**
Age, mean (SD)	37.23 (7.18)	37.07 (7.33)	*P* = 0.878
Highest education level, % (*n*)			
Low (less than high school)	25.3 (44)	20.4 (135)	*P* = 0.091
Medium (high school /trade/diploma)	49.4 (86)	46.2 (306)	
High (tertiary)	25.3 (44)	33.5 (222)	
Born in Australia, % (*n*)	90.8 (158)	92.2 (611)	*P* = 0.671
Employment status, % (*n*)			
Full-time	39.7 (69)	36.7 (243)	*P* = 0.146
Part-time	29.3 (51)	37.1 (246)	
Not currently employed	31.0 (54)	26.2 (174)	
Relationship status, % (*n*)			
Married/de facto	70.1 (122)	76.3 (506)	*P* = 0.188
Separated/divorced/widowed	8.0 (14)	7.5 (50)	
Never married	21.8 (38)	16.1 (107)	
Area of residence, % (*n*)			
Urban	44.8 (78)	39.5 (262)	*P* = 0.237
Rural	55.2 (96)	60.5 (401)	
Smoking status, % (*n*)			
Never smoked	46.6 (81)	56.0 (371)	***P*** **= 0.035**
Former smoker	29.3 (51)	27.1 (180)	
Current smoker	24.2 (42)	16.9 (112)	
BMI categories, % (*n*)			
Under/healthy weight	50.6 (88)	56.9 (377)	***P*** **= 0.015**
Overweight	25.3 (44)	28.2 (187)	
Obese	24.1 (42)	14.9 (99)	
BMI, mean (SD)	26.38 (6.09)	25.45 (5.22)	*P* = 0.108
Total minutes per week leisure time physical activity, mean (SD)	221.90 (295.98)	220.98 (286.47)	*P* = 0.717

To place the present findings in context against current population dietary recommendations and norms, the diets of the READI women included in this study (as measured by individual food group consumption at baseline) were compared with the Australian dietary guideline recommendations and population dietary intake data (Table 4). At baseline, the majority of women were consuming diets of poor nutritional quality (> 90% of the cohort failed to meet the recommended guidelines for vegetables, grains, lean meat and alternatives, and dairy foods). For the five key food groups, the diets of READI women were highly comparable to Australian population norms (Table 4).

**Table 4:** Dietary intake of READI participants at baseline, and AGHE recommended serves per day

	AGHE recommended serves per day	Australian population data intake (women aged 19 – 50 yrs)	READI Cohort (*n* = 837)
Vegetables (serves per day), mean	5	2.5^c^	2.82
Meets vegetable guidelines, %	–	10^c^	6.6
Fruit intake (serves per day), mean	2	1.8^c^	1.64
Meets fruit guidelines, %	–	55^c^	51.3
Grains intake (serves per day), mean	6	3.8^d^	3.12
Meets grains guidelines, %	–	8.5^d^	3.3
Lean meats intake (serves per day), mean	2.5	1.6^d^	1.51
Meets lean meats guidelines, %	–	5.3^d^	6.8
Dairy intake (serves per day), mean	2.5	1.3^d^	1.40
Meets dairy guidelines, %	–	6.0^d^	9.7
Discretionary items (serves per day), mean^f^	≤ 2.5	3.2^e^	0.85
Sugars (serves per day), mean			0.51
Meets discretionary items^b^, %	–	–	96.8
Meets sugars^b^, %	–	–	92.4
Meets alcohol guidelines^b^, %	≤ 2	90.7^c^	84.6

^a^ Meets guidelines – achieves the maximum DGI score for that dietary component

^b^ Indicates the proportion of individuals who *did not exceed* the recommended serves / day

^c^ National Health Survey: First Results [44]

^d^ Australian Health Survey: Consumption of food groups from the Australian Dietary Guidelines [45]

^e^ Presented as median. Mishra et al., 2014 [46]

^f^ READI women are shown to be consuming fewer discretionary items than population figures. This may be related to the fact that the Australian Health Survey data included added sugars and alcohol within the discretionary item category, which was not the case for this study

### Diet quality and depressive symptoms

The generalized linear model provided evidence of a relationship between baseline diet quality and risk of developing depressive symptoms at 5 years follow-up, where a 10 unit increase in DGI-2013 score (of a possible 85) was associated with 12% lower risk of developing heightened depressive symptoms (RR for 10 unit increase = 0.875, 95% CI 0.784 to 0.978, *p* = 0.018). This relationship was evident whilst adjusting for age, highest education level, country of birth, employment status, relationship status, location of residence, total leisure-time physical activity, BMI, smoking status and clustering by neighbourhood.

The fixed-effects model indicated an inverse relationship between concurrent change in diet and depressive symptoms, such that an increase in DGI score over 5 years follow-up was associated with a lower (improved) CES-D score (B = -0.044, 95% CI − 0.08 to − 0.01, *p =* 0.024). Thus, for example, a 10-unit increase in DGI score for a person over time would correspond with an estimated decrease of 0.44 in CESD-D score.

## Discussion

This study demonstrated that adherence to the Australian Dietary Guidelines was associated with a reduced risk of developing depressive symptoms at 5-years follow-up amongst women recruited from socio-economically disadvantaged neighborhoods in Australia. Every 10 unit increase in DGI-2013 score (of a possible 85) was associated with a 12% reduced risk of developing depressive symptoms. These findings are consistent with those of other longitudinal studies, which have shown that consumption of anti-inflammatory diets (high in nutrients such as omega-3 and omega-6 polyunsaturated fatty acids, dietary fibre and food groups such as fruits, vegetables, meat, red meat, fish and high-fibre grain foods) [47], and better adherence to three diet quality scores (Mediterranean Diet Score (MDS), Pro-Vegetarian Dietary Pattern (PDP), and Alternative Healthy Eating Index-2010 (AHEI-2010) were associated with a 20 to 40% reduced risk of developing depression [48]. Additionally, the Whitehall II Study found that the AHEI score was inversely associated with recurrent depressive symptoms in a dose-response fashion, and that women who maintained high AHEI scores had 65% lower odds of subsequent recurrent depressive symptoms compared with women who maintained low AHEI scores over 10 years [49]. Only recently, the world’s first RCTs to show an effect of dietary patterns on mental health in adults with depression were published. The HELFIMED study reported that a Mediterranean-style diet supplemented with fish oil improved mental health and depressive symptoms compared to control group over 3 months (with results also sustained at 6 months) in 152 adults with depression [18]. Additionally, “SMILES” showed remarkably similar results over 12 weeks in 67 adults diagnosed with major depressive disorder, and remission of depression was achieved for 32.3 and 8.0% of the intervention and control groups, respectively [17]. These studies highlight the consistency of findings regarding the association between diet and depression, despite the differing dietary patterns explored and methodological approaches used. Furthermore, the present study extends these findings to women of low SEP who are a high risk population with greater likelihood of depression [4] and poorer adherence to dietary guidelines [10].

In terms of practical dietary changes required to achieve a 10 unit increase in DGI score, a person would need to have approximately 0.5 additional serves of fruit per day (= 2.5 points), 0.6 additional serves of dairy per day (e.g. ~ 2/3 cup of milk or 120 g yoghurt) (= 2.5 points) and change from white bread to wholemeal/multigrain bread (= 5 points). Such dietary changes are likely to be feasible and realistic. For example, Australian nutrition promotion campaigns have achieved a net increase of 0.8 mean number of servings of fruit and vegetables per day [50], while international initiatives have observed impacts on fruit and vegetables ranging from 0.2 to 0.7 portions for fruit and vegetable consumption across different income groups in the UK [51], and 0.2 to 1.1 portions per day among the general or low income populations in the US [52]. These findings also demonstrate potential benefits of targeted campaigns for lower socioeconomic groups specifically, like the present sample.

Our results are encouraging in terms of suggesting that even small improvements in diet quality may result in a reduced risk of developing depressive symptoms. This is particularly important for socio-economically disadvantaged individuals who have less disposable income and face other challenges to adhering to dietary guidelines [53]. The women included in this study had typically poor quality diets, which were reflective of intakes of the Australian population [35] and the majority of Australian women have significant scope for improvement in meeting the dietary guidelines [46]. Moreover, the socio-demographic characteristics of the study population were comparable to population norms, and thus likely generalisable. This data adds to growing evidence attesting to the importance of population dietary shifts of even small magnitudes. For example, it has been estimated that if vegetable consumption in Australia was only 10% higher, the government health expenditure would reduce by $99.9 million (in 2015–16 dollars) [54].

Our study aimed to examine associations in a disadvantaged population, given their increased risk of both poor diet and depression. Whether such findings would hold in other samples such as those of higher SEP is unknown, though likely if biological rather than social mechanisms are responsible for diet-depression links. While determining the mechanisms underpinning diet-depression associations was beyond the scope of this study, evidence indicates multiple pathways by which foods and nutrients may impact on mental health [55]. Acknowledging that the cohort study design cannot unequivocally determine a causal effect, the improvements in depressive symptoms observed here may result from the cumulative and synergistic effects of the foods and nutrients that comprise the healthy dietary pattern in its entirety, rather than from the effects of individual nutrients or single foods. This healthy dietary pattern is characterised by an adequate intake of the following nutrients with known beneficial health effects; omega-3 PUFAs (from fish), dietary fibre and antioxidants (e.g. vitamins A, E and ß-carotene) (from legumes, fruits, vegetables and whole grains), and a limited intake of discretionary items (e.g. sweets, highly processed cereals, crisps, fast-food, sugary drinks), which are typically high in harmful trans-fatty acids, SFAs, refined carbohydrates and added sugars [56]. Potential biological pathways related to depression (and likely modulated by diet) include inflammation, oxidative stress, the gut microbiome, epigenetic modifications and neuroplasticity [55–57].

### Strengths and limitations

A potential study limitation is that only 19% of the original READI sample were eligible for analysis, and the included sub-sample were of a higher socioeconomic position and more likely to report healthier behaviours than the remaining READI sample, which may limit generalisability. However, as mentioned above, the diets and socio-demographic characteristics of this sub-sample were comparable to population norms. A further limitation is that measures of food intake were self-reported, which tends to result in under-reporting among women [58, 59]. As the current study used a brief FFQ, which did not provide an overall measure of energy intake, we were unable to adjust for total energy intake or assess for energy misreporting [59]. However, there is evidence that restricting samples or adjusting analyses on the basis of energy misreporting can itself be problematic, leading to selection bias or inflated associations with outcomes [60]. In addition, the diet quality score takes into account variations in energy intake as it uses age- and sex-specific cut-offs for intake of food, and our statistical analysis adjusted for key determinants of energy intake such as age and physical activity. While certain reported dietary intakes (particularly for discretionary foods) were lower in this study than in the general population [44] the FFQ was based on validated measures, and used to calculate a food-based diet quality score, which has been shown to be associated with intakes of key nutrients and health outcomes [33, 35–37]. The DGI used was a modified (non-validated) version, which encompassed fewer food components, and hence may not provide an accurate representation of nutritional adequacy. Importantly, however, the modified DGI did retain the key elements of the Australian Dietary Guidelines (e.g. all five food groups and discretionary foods were included). The impact of modifying this score is unknown, and a common concern within the field of diet quality scores, but could be the focus of future methodological work. Finally, a number of plausible confounders that could potentially explain the relationship between diet quality and depressive symptoms (e.g. antidepressant medication use, sleep quality, comorbidities such as cancer or cardiovascular disease) were not analysed in this study.

A strength of this study was the large sample size which allowed for controlling of relevant confounders, including BMI. Depression has been shown to be predictive of the development of overweight and obesity and, in turn, overweight and obesity are predictive of the development of depression [61]. In this study, the association between diet and depressive symptoms remained evident even after adjusting for BMI, which is an important finding as it suggests that the relationship was likely explained by diet per se. The relationship between diet and mental health is complex and likely bidirectional [62]. For example, a change in dietary intake may occur in response to the altered appetite and cravings (or a reduced interest in food) associated with clinical depression and depressive symptoms [63–65]. Alternatively, depression is commonly associated with fatigue and apathy [66], which may impact on an individual’s motivation to engage in healthy dietary habits [67], a reduced desire to cook and prepare meals [68], and depleted energy for activities such as food shopping and meal preparation [69, 70]. A major strength of this prospective study design was the capacity to demonstrate the expected direction of relationship between habitual diet and development of depressive symptoms. Furthermore, to address the possibility of reverse causality, women with reported depressive symptoms at baseline were removed from the analysis. While the prospective design and exclusion of those with baseline symptoms is useful for establishing temporality, it does not conclusively establish causality and we cannot rule out residual confounding. Ideally these results would be further confirmed using RCT approaches.

## Conclusions and potential implications

This prospective study demonstrated that adherence to a healthy diet was associated with reduced risk of development of depressive symptoms. A dietary approach for the prevention and management of depression, with negligible risks and side effects associated, may be of considerable interest to practitioners and society. There is a strong overlap between depression and cardiometabolic conditions (e.g. obesity, insulin resistance, metabolic syndrome, diabetes and CVD), where up to 20–25% of individuals with cardiometabolic and other chronic medical conditions will develop major depressive disorders [71]. Moreover, the association is bidirectional, and is likely mediated through multiple mechanisms [72]. Thus, behaviour changes, such as adopting a healthy diet, that have considerable breadth to target both depression and cardiometabolic diseases may achieve substantial reductions in disease burden and savings to the healthcare system [73]. Our results are encouraging in terms of suggesting that women from socioeconomically disadvantaged neighbourhoods who have diets that align more closely with the Australian Dietary Guidelines are at a reduced risk of developing depressive symptoms. The diet quality of the Australian population continues to be poor [35], and yet there are many practical strategies for addressing food behaviours that contribute to improved diet quality. The growing high-quality evidence regarding the relationship between diet and depressive symptoms provides us with a strong rationale for developing scalable strategies for supporting dietary behaviour change programs to assist in lowering depression rates.

## Supplementary information

**Additional file 1: Supplemental Table 1** Components of the dietary guideline index.

## Data Availability

The datasets used and/or analysed during the current study are available from the corresponding author on reasonable request and subject to appropriate ethical approvals being granted.

## Abbreviations

AGHE: Australian guide to healthy eating
AHEI-2010: Alternative healthy eating index-2010
BMI: Body mass index
CES-D: Centre for epidemiologic studies depression
CVD: Cardiovascular disease
DGI-2013: Australian dietary guideline index
FFQ: Food frequency questionnaire
HEI: Healthy eating index
IPAQ: International physical activity questionnaire
IRR: Incidence risk ratios
MDS: Mediterranean diet score
PDP: Pro-vegetarian dietary pattern
PUFAs: Polyunsaturated fatty acids
RCTs: Randomized controlled trials
READI: Resilience for eating and activity despite inequality
SEP: Socio-economic position
SFAs: Saturated fatty acids
