# Supplementary material for: Adherence to the Australian dietary guidelines and development of depressive symptoms at 5 years follow-up amongst women in the READI cohort study
Source: Nutr J. 2020 Apr 10;19:30. doi: 10.1186/s12937-020-00540-0 (PMC7149932; doi:10.1186/s12937-020-00540-0)
Supplement: Supplementary file 1 — Additional file 1: Supplemental Table 1 Components of the dietary guideline index. [file 12937_2020_540_MOESM1_ESM.docx]

**Supplemental Table 1** Components of the dietary guideline index ^(1)^

| **Dietary Guideline** | **Indicator and Description** | **Criteria for maximum score** ^a^ | **Maximum score** ^a^ | **Criteria for minimum score** ^a^ | |
| --- | --- | --- | --- | --- | --- |
| 1. Plenty of vegetables | Total vegetable intake (including potatoes): servings of vegetables per day | ≥ 5 | 10 | 0 | |
| 2. Fruit | Total fruit intake: servings of fruit per day ^c^ | ≥ 2 | 10 | 0 | |
| 3. Grain (cereal) foods | Total cereal intake: servings of grains per day | ≥ 6 | 5 | 0 | |
|  | Mostly wholegrain or high fibre cereals: type of bread usually consumed | Wholemeal bread, multigrain bread, rye bread | 5 | White bread | |
| 4. Lean meat and poultry,  fish, eggs, nuts and seeds,  and legumes/beans | Total meat and alternative:  servings per day | ≥ 2.5 | 5 | 0 | |
| 5. Milk, yoghurt, cheese  and/or their alternatives | Total dairy and alternative:  servings per day. Includes flavoured milk. | ≥ 2.5 | 10 | 0 | |
| 6. Limit intake of foods  containing saturated fat,  added salt, added sugars  and alcohol ^b^ | Limit discretionary foods daily. Includes hot chips; potato crisps or salty snacks; cake, doughnuts, sweets biscuits; pies, pasties or sausage rolls; fast foods; pizza; meat products (e.g. sausages). | ≤2.5 | 10 | > 2.5 | |
| 7. Limit intake of foods  high in saturated fat | Trim meat: trimming fat  from meat | Usually, always | 5 | Never, rarely |  |
|  |  | Sometimes (score of 1.25) |  |  |  |
|  | Choose reduced-fat milk: type of milk usually consumed | Skim, low or reduced  fat milk, soy | 5 | Whole milk | |
| 8. Limit intake of foods  and drinks containing  added sugars^b, c^ | Limit extra sugar: servings per day. Includes soft drink, fruit juice >125mL per day, chocolate or lollies. | ≤1.25 | 10 | > 1.25 | |
| 9. If you choose to drink  alcohol, limit intake ^d^ | Limit alcohol: servings per day | ≤ 2 | 10 | > 2 | |

^a^ Based on recommendations from the Australian Guide to Healthy Eating for women aged 19-50 years ^(2)^. Servings unless otherwise indicated. Participants with intakes between the maximum and minimum amount were assigned scores proportionately.

^b^ Guidelines for added sugars and discretionary foods are presented as an upper limit. As there is no quantitative guideline for added sugars, half the maximum discretionary foods cut-off was used which is consistent with existing dietary indices ^(3)^

^c^ The fruit group does not include fruit juice as it was not possible to determine whether the fruit juice had no added sugars due to the lack of information available in the FFQ used in this study. The Australian Guide to Healthy Eating currently allows ≤125mL of fruit juice with no added sugars “occasionally”. Subsequently, fruit juice intake of >125mL was included in the added sugars group.

^d^ The scoring of Alcohol was categorical in nature, where a score of ten was awarded for remaining below the cut-off (≤ 2 glasses per day) or zero for exceeding it (> 2 glasses per day). The 2009 National Health and Medical Research Council (NHMRC) guidelines for reducing health risks associated with the consumption of alcohol state that, for healthy men and women, 'drinking no more than two standard drinks on any day reduces the lifetime risk of harm from alcohol-related disease or injury' ^(4)^.

**References**

1. Thorpe MG, Milte CM, Crawford D, McNaughton SA. A Revised Australian Dietary Guideline Index and Its Association with Key Sociodemographic Factors, Health Behaviors and Body Mass Index in Peri-Retirement Aged Adults. Nutrients. 2016;8:160.

2. National Health and Medical Research Council. The Australian Dietary Guidelines. Healthy eating for adults: eat for for health and wellbeing. NHMRC: 2013.

3. McNaughton SA, Ball K, Crawford D, Mishra GD. An Index of Diet and Eating Patterns Is a Valid Measure of Diet Quality in an Australian Population. J Nutr. 2008;138:86-93.

4. National Health and Medical Research Council. Australian guidelines to reduce health risks from drinking alcohol. NHMRC, 2009.
